# Introduction of West Nile virus lineage 2 leads to neuroinvasive cases in humans and horses in Sicily, 2022–2023

**DOI:** 10.1186/s13567-025-01575-z

**Published:** 2025-09-25

**Authors:** Francesca Gucciardi, Simona De Grazia, Annalisa Guercio, Francesco La Russa, Maria Liliana Di Pasquale, Floriana Bonura, Federica Monaco, Massimo Spedicato, Barbara Bonfini, Giovanni Savini, Giovanni M. Giammanco, Giuseppa Purpari

**Affiliations:** 1 Istituto Zooprofilattico Sperimentale della Sicilia “A. Mirri”, Via Marinuzzi, Palermo, Italy; 2 Department of Health Promotion, Mother and Child Care, Internal Medicine and Medical Specialties “G. D’Alessandro”, University of Palermo, Via del Vespro 133, Palermo, Italy; 3 Istituto Zooprofilattico Sperimentale dell’Abruzzo E del Molise, Teramo, Italy

**Keywords:** West Nile virus, lineage 2, Italy, neuroinvasive

## Abstract

In Italy, West Nile virus (WNV) has been consistently causing disease in humans, horses, and birds since 2008; it was initially confined to the northeast of the country and then spread gradually to the western and southern regions. WNV lineage 1 (WNV-L1) virus was the only lineage that circulated in Italy until 2011, but it was progressively replaced by WNV lineage 2 (WNV-L2). WNV disease in humans was not observed in Sicily, southern Italy, until 2016, when the first case of West Nile neuroinvasive disease (WNND) due to WNV-L1 was reported. Our results demonstrate the introduction of WNV-L2 in Sicily via the detection of four equine and five human cases of WNND reported in western Sicily in September 2022. Local WNV circulation was confirmed by virological and serological investigations in horses, birds, and dogs. WNV-L2 was demonstrated to be the strain responsible for both human and equine neuroinvasive cases and continued to circulate in western Sicily in 2023, where it was detected in insects and in a neuroinvasive human case in August.

## Introduction

Emerging and re-emerging vector-borne diseases are major public health threats worldwide, causing more than 1 million human deaths annually [1]. Among these diseases, West Nile disease (WND), caused by West Nile virus (WNV), is the most widespread vector-borne disease in the world and causes disease and death in humans, birds and equids [2]. WNV lineages 1 (WNV-L1) and 2 (WNV-L2) are by far those most widespread in Europe and the Mediterranean region [3]. WNV is an RNA virus within the *Orthoflavivirus nilense* species recently included in the *Orthoflavivirus* genus, family *Flaviviridae*. WNV is transmitted mainly by *Culex* sp. mosquitoes and can infect humans, birds, horses, and other mammals, causing severe disease and death. The virus is maintained in nature in an enzootic cycle involving competent mosquitoes and a wide variety of reservoir bird species, which act as amplifying hosts [4]. Accidentally, the virus can infect mammals, which are dead-end hosts, as infections do not produce a sufficiently high level of viraemia to retransmit the virus to competent vectors [5]. In humans, infections are mostly asymptomatic, and in approximately 20% of cases, WNV infection causes a mild disease with influenza-like symptoms termed West Nile fever (WNF), whereas in less than 1% of cases, especially in elderly and immunocompromised people, infections can lead to severe neuroinvasive disease (WNND), which may be fatal [6]. Horses are more susceptible to WNV than humans are, as up to 10% of infected horses may show neurological signs [7, 8]. The first human outbreak of WNND in Europe occurred in Romania in 1996, with 393 confirmed cases [9]. In recent years, the number of WNND human cases has increased significantly, especially in Europe and the Mediterranean Basin, where it peaked in 2018 [10–12]. In Italy, WNV has caused severe disease in humans, horses and birds, starting from northeastern Italy and then spreading gradually to the western and southern parts of the country. The first finding of WNND in Sicily occurred in August 2010, with 5 horses showing neurological symptoms in the Trapani district. The following year, WNV-neutralizing antibodies were detected in dogs living in the city of Trapani, confirming the circulation of the virus in urban areas [13, 14]. A few other horses were observed in 2011 in the district of Messina and around Palermo [15]. After the first case of WNND in Sicily in 2016, in the Trapani district [16], no more cases of human or animal WNV infection were reported. Initially, WNV-L1 was the only lineage circulating in Italy; then, (from 2011), it was progressively replaced by WNV-L2 [17]. In the last three years, both lineages have cocirculated in most parts of the country [18–20]. The WNV National Italian Surveillance Program, which is coordinated by the Ministry of Health and aims to detect the introduction of WNV and monitor its circulation throughout the territory, has been operative since 2001. First, it was based only on entomological surveillance and serological screening of sentinel horses and poultry, but since 2016, the plan has been integrated with data from WNV and WNND in humans [1, 21, 22]. In 2020, to strengthen and integrate the activities and collaboration between various professional figures from a “One Health” perspective, the Ministry of Health developed the National Plan for Prevention, Surveillance and Response to Arboviruses (PNA) 2020–2025, which is divided into a six-year time frame, which provides integrated veterinary, entomological and medical surveillance aimed at early identification of arbovirus circulation in the national territory in birds or insect vectors to promptly implement all available measures to prevent transmission to humans through targeted long- and medium-term programs [1]. According to the latest guidelines from the Italian Ministry of Health, WNV surveillance is performed throughout the year by Regional Reference Laboratories, and its intensity in various areas depends on the risk of WNV circulation (areas of high, low and minimal risk of transmission). In high- and low-risk areas, the plan is based on the following: surveillance of stationary birds belonging to target species (in low-risk areas, it is possible to implement surveillance on rural or open-air poultry farms); surveillance of specimens of dead wild birds; entomological surveillance; clinical surveillance of equines; and surveillance of human cases. In areas with a minimum risk of transmission, entomological and stationary bird surveillance is not carried out. During veterinary surveillance, a case of WND is considered confirmed when there is seroneutralisation positivity in subjects from rural or free-range poultry farms; positive IgM ELISA and/or molecular tests (RT‒PCR) in equidae with clinical symptoms attributable to WND; RT‒PCR positivity in the organs or blood of birds (sampled or found dead); and RT‒PCR positivity in mosquito pools. A human case of WND is confirmed when neurological symptoms or a febrile illness are reported in association with at least one of the following laboratory criteria: WNV isolation or WNV RNA detection in blood, urine or cerebrospinal fluid (CSF); presence of specific anti-WNV IgM in CSF; high IgM antibody titre; and IgG detection in serum. The specificity of IgG must be confirmed in both human and animal cases via neutralization assays to define WNV antibody titres and to exclude any cross-reaction with other cocirculating *Flaviviruses*, such as Usutu. Confirmation tests are carried out by National Reference Laboratories, Istituto Superiore di Sanità (ISS), Rome, for human samples, and Istituto Zooprofilattico Sperimentale dell’Abruzzo e del Molise (IZS-Te), Teramo, for vector and animal samples. When a WND case is confirmed, surveillance activities in the area should be intensified by conducting an epidemiological survey in reservoir birds, animals and vector susceptible species close to the presumed exposure site of the cases to assess viral circulation and activate specific control measures on donations of SoHO (substances of human origin), such as blood and blood components, organs and tissues. In 2022, 949 human cases of WNV infection were reported in EU member states. In Italy, the national surveillance plan reported 588 human cases and 37 deaths due to WNV infection [10, 23, 24]. Since the beginning of the 2023 transmission season and as of 6 December 2023, EU countries have reported 707 human cases of WNV infection, with 336 cases and 29 deaths in Italy [25].

According to the risk-based classification, Sicily has been considered a high-risk area because of neurological cases reported in horses in 2010 [13]. A human case of WNND was reported in 2016 [16]. No more cases have been observed in the region since then. This study provides a picture of the 2022–2023 WNV vector season in western Sicily and describes WND cases detected in humans and animals.

## Materials and methods

### WNV human investigation

During the 2022–2023 epidemic seasons, a total of 66 patients (ranging between 13 and 84 years old, average age 54.4) from western Sicily showing neurologic symptoms were investigated by the Microbiology and Virology Unit at the AOUP “Paolo Giaccone” University Hospital of Palermo. In particular, 33 patients were tested for WNV between 29 August and 20 December 2022, and 33 were tested between 9 February and 1 December 2023.

### Laboratory diagnosis

A total of 60 serum samples, 29 from 2022 and 31 from 2023, were screened for the presence of anti-WNV IgM/IgG by a commercial enzyme-linked immunosorbent assay (ELISA) (WEST NILE VIRUS VIRCLIA® IgM and IgG, Vircell). According to the integrated national program guidelines, a positive case was confirmed by the detection of a high titre of WNV IgM and/or WNV IgG confirmed by a specific plaque reduction neutralization assay (PRNT) [26], which was performed at the ISS or by the detection of WNV nucleic acid in the blood or CSF. The WNV genome was searched in a total of 28 CSF, 35 whole blood and 19 urine samples collected from 66 Sicilian patients with neurological symptoms. WNV RNA was extracted from CSF, blood and urine samples via the ELIte InGenius extraction kit, CE-IVD, according to the manufacturer’s instructions. The extracted RNA was examined by one-step quantitative multiplex real-time reverse transcription polymerase chain reaction (qRT‒PCR) (WNV ELITe MGB Kit, Elitech Group), which allows the simultaneous detection of WNV lineages 1 (L1) and 2 (L2) from whole blood samples collected in EDTA, CSF, and urine collected without preservatives. The qRT‒PCR test amplified a region of the NS5 gene of WNV, encoding a nonstructural protein, and a region of the genomic RNA of the MS2 phage was used as an internal process control (IPC) for nucleic acid extraction.

### Veterinary surveillance

In 2022, after the confirmation of human cases of WNND, ad hoc active entomological and animal monitoring programs were implemented in the area surrounding the homes of the human cases (within a radius of up to 20 km). In the Trapani district, the program was activated on 22 September 2022, following confirmation of the first WNND human case in Sicily, recorded on 13 September. In the bordering areas of the Palermo district (Partinico area), the program was activated on 24 October 2022, after WNND was detected in a horse. Animals (the serum and/or blood of dogs, equids and birds) and entomological samples collected for the monitoring program were investigated at the Istituto Zooprofilattico Sperimentale della Sicilia (IZSSi).

On 23 August 2023, a pool of mosquitoes from the province of Trapani tested positive for WNV and were from the same area where a case of WNND was confirmed in a 54-year-old immunocompromised man hospitalized with encephalitis. These events led to an intensification of the surveillance activities required by the PNA.

### WNV entomological investigation

BG-Sentinel, CDC-light and gravid traps were placed in the Trapani and Palermo areas for entomological monitoring. The trap placement sites were established by identifying potentially favourable habitats for the larval and adult stages of the insects and recording the georeferencing. The traps were placed outside under sheds or other coverings, such as porches, which were not exposed to prevailing winds, were protected by the sun and had a maximum height of 2 m. The trapping area is characterized by a semirural environment near small family-run poultry or horse farms.

**Figure 1:**
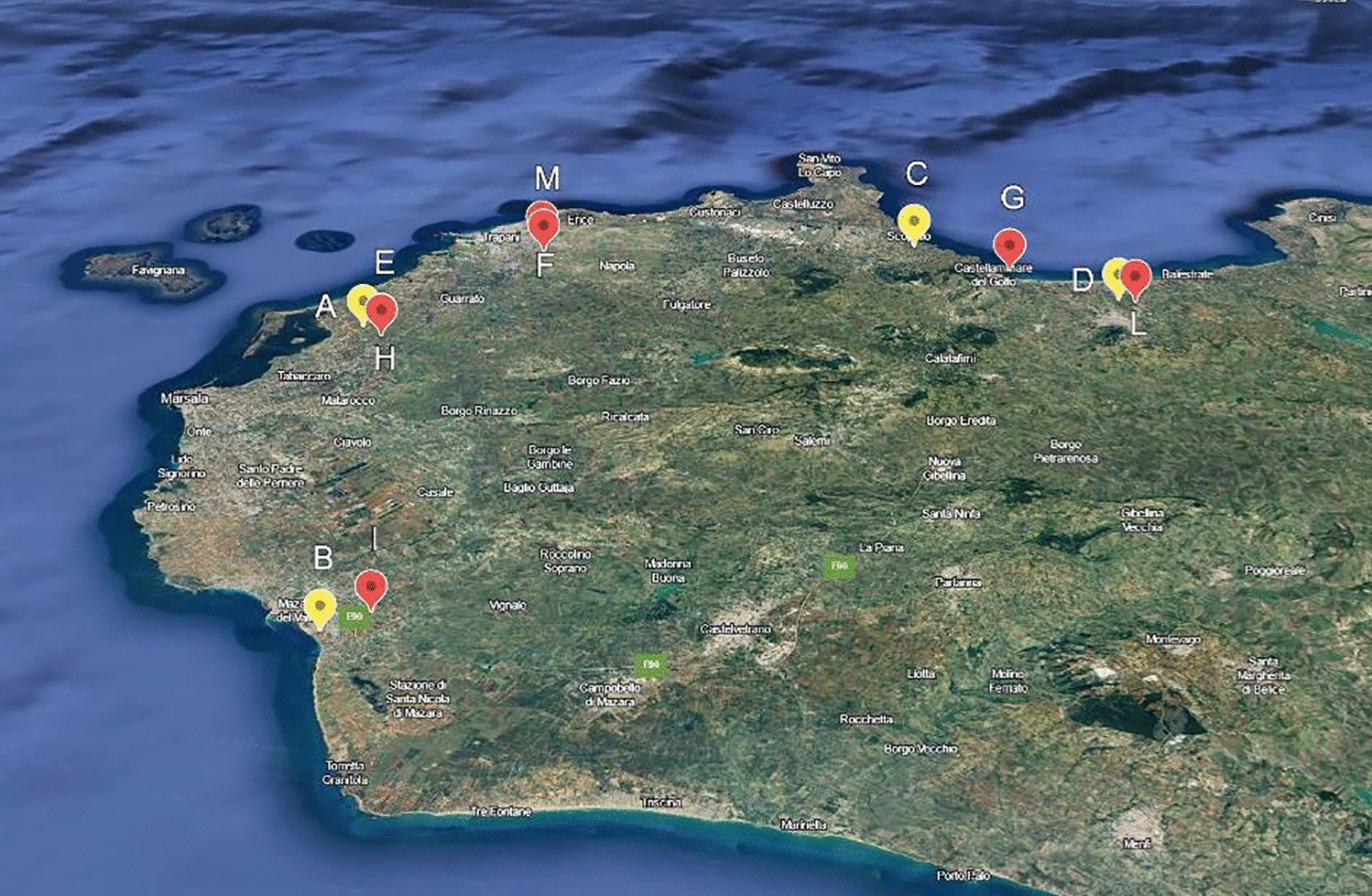
**Sites of entomological investigations monitored between 2022 (yellow) and 2023 (red)**.

In 2022, the entomological traps were positioned at five collection sites (Sites A–E) (Figure 1) and were periodically sampled every fifteen days between September and October. The WNND-positive man house, situated in a suburban area near the lagoon of the Stagnone of Marsala (a humid area, natural reserve, including some coastal ponds, ideal habitat for migratory birds), corresponded to Site A. The other traps were located in areas neighbouring equine cases of WNND, at a distance of less than 20 km, in potential larval breeding sites, such as cisterns, canals, artificial drains, ponds and gardens (Table 1). In 2023, there were six trap placement sites in the province of Trapani, five of which fell within the same municipalities as those in the previous year (Sites F–L) and one near 2023 human clinical cases and positive mosquitoes (Site M) (Figure 1, Table 1).

**Table 1:** **Entomological collection sites, with geographical coordinates, altitudes, and main environmental and positioned trap characteristics**.

	Geographic coordinates				Traps	
	Latitude	Longitude	Altitude	Description	years	Number/Kind
Site A	37,862,250	12,509,250	167	Near human clinical case home	2022	7/CDC, Gravid traps, BG Sentinel
Site B	37,639,101	12,611,357	344	Within 20 km from 1 symptomatic horse	2022	2/BG Sentinel
Site C	38,046517	12,825,591	164	Farm with 1 symptomatic horse	2022	8/CDC, Gravid traps, BG Sentinel
Site D	37,993,345	12,962,820	344	Farm with 1 symptomatic horse and near to 1 other symptomatic horse	2022	3/CDC, Gravid traps
Site E	37,872,126	12,521,745	316	Neighbouring site A	2022	1/BG Sentinel
Site F	38,003250	12,573,440	240	Positive mosquito near 2023 human clinical case home	2023	4/BG Sentinel
Site G	38,019054	12,892,416	438	Neighbouring site C and D	2023	1/BG Sentinel
Site H	37,872,126	12,521,745	276	Neighbouring site A and within 20 km from site F	2023	1/BG Sentinel
Site I	37,655,400	12,627,399	220	Neighbouring site B	2023	1/BG Sentinel
Site L	37,995,600	12,973,440	266	Neighbouring site D	2023	3/BG Sentinel
Site M	37,967,977	12,594,041	321	Near site F	2023	4/BG Sentinel

### Laboratory diagnosis

**Figure 2:**
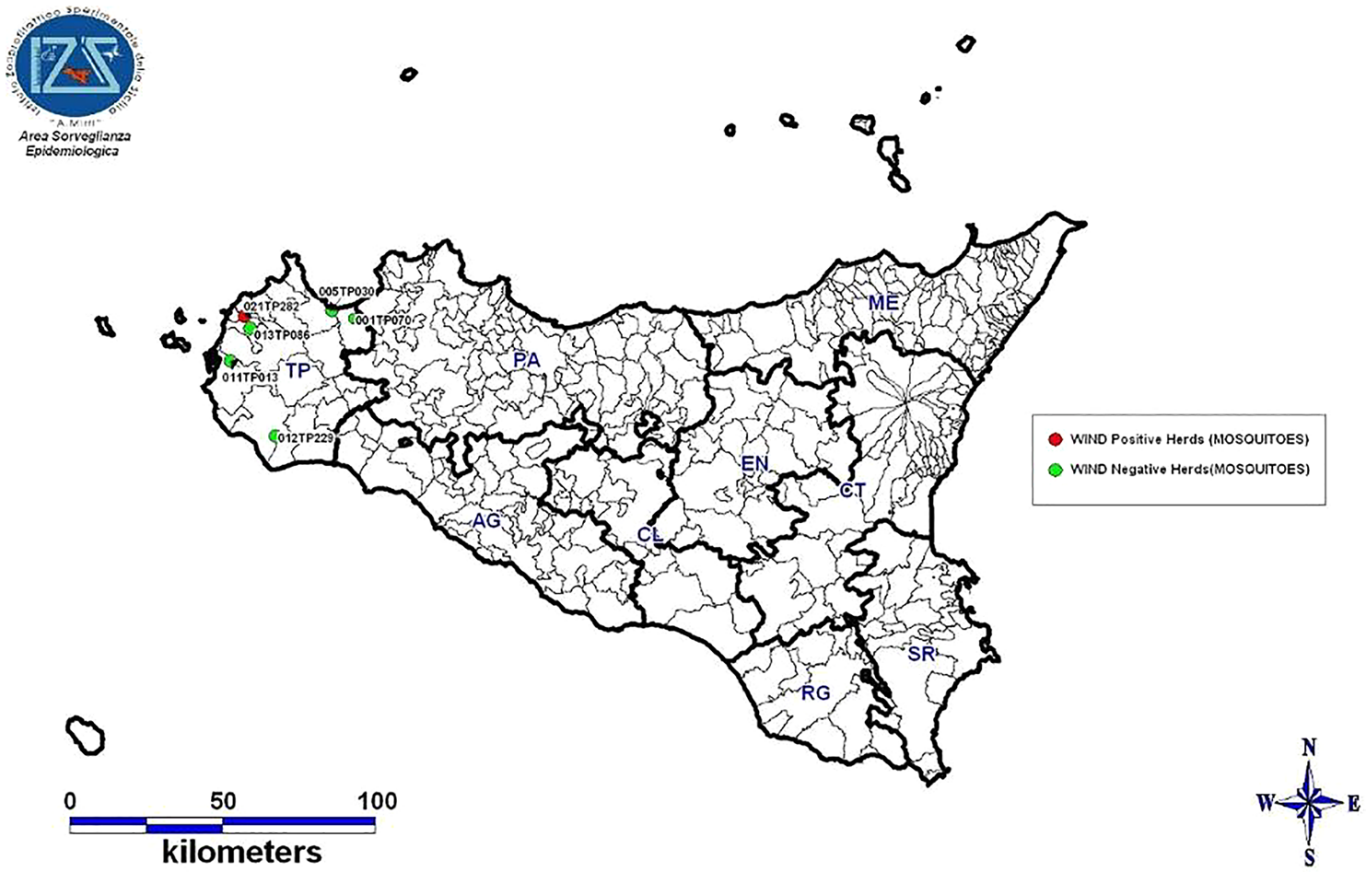
**Geolocation of farms where mosquito traps were placed in 2023**.

The sampled mosquitoes were morphologically identified at the species level by stereomicroscopy [27]. The collected mosquitoes were pooled according to species, collection site and date sampling. In 2022, up to 60 samples were pooled. However, only one pool included 60 mosquitoes since the number of insects caught per trap was, in most cases, much lower (less than 21). In total, 35 pools were obtained from the Trapani area (with 1–60 mosquitoes per pool), and 4 pools were obtained from the Partinico area (with 1–9 mosquitoes per pool). Between 23 August and 25 October 2023, 14 pools of mosquitoes (with 1 to 22 mosquitoes per pool) were obtained from six locations in the Trapani area (Figure 2).

Pools of mosquitoes were homogenized in phosphate-buffered saline (PBS) (pH 7.2) supplemented with antibiotics. Viral RNA was extracted by using a commercial kit (QIAamp Viral RNAMini Kit, QIAGEN) according to the manufacturer’s instructions.

The presence of WNV was detected by multiplex qRT‒PCR for the simultaneous detection of WNV-L1 and WNV-L2, including an IPC (targeting the NS5-2 region of WN-HNY99), using the Quantitect Probe RT‒PCR Kit (QIAGEN) [16, 29].

### WNV animal investigation

According to the animal monitoring programs, 11 dogs, 76 equids (4 with neurological symptoms), and 66 birds (64 chickens and 2 geese) were sampled in 2022 in the district of Trapani, whereas 22 horses (1 with neurological symptoms) were sampled in the Palermo district (Figure 2). All symptomatic horses recovered except for one animal from the Palermo district, which died 9 days after the onset of clinical signs.

In 2023, 5 farms from five different municipalities in the Trapani district were monitored. Apart from one that also had sheep, all the farms included in the surveillance program housed only horses. No clinical signs were observed in any of the animals from these farms. Therefore, no samples were collected.

### Laboratory diagnosis

**Figure 3:**
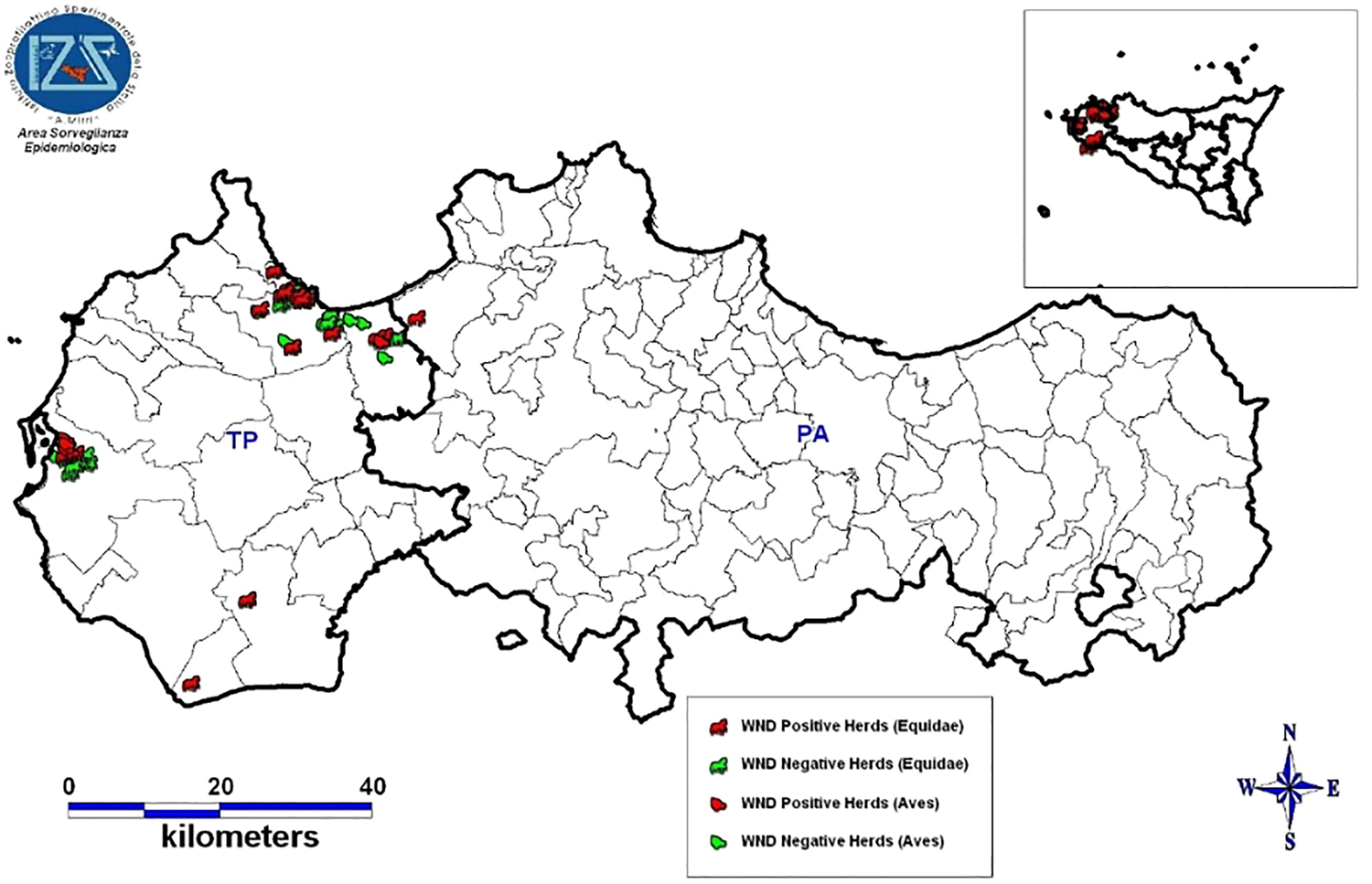
**Geolocation of *Aves* and *Equidae* farms investigated in 2022.**

In 2022, 98 serum samples from horses were tested for the presence of WNV IgM (ID® Screen West Nile IgM Capture, ID. Vet) and WNV IgG (ID Screen West Nile Competition Multispecies, ID-Vet Innovative Diagnostics, Grabels, France). Similarly, serum samples from birds (*n* = 66) and dogs (*n* = 11) were tested for the presence of WNV IgGs and confirmed by a serum neutralization test in microtiter format [28]. WNV and lineage-specific qRT‒PCR [29] were performed on the RNA extracted from the blood samples of 22 horses and 66 birds from Trapani and 20 horses from the Palermo district. Virological tests were also performed on the brain, spinal cord and medulla oblongata of the one horse that died after showing neurological signs (Figure 3).

## Results

### WNND human cases

In the 2022 season, the Microbiology and Virology Unit of the “Paolo Giaccone” University Hospital AOUP of Palermo detected high levels of anti-WNV IgM in five patients originating from western Sicily, three adults from Trapani (26, 69 and 72 years old) and two from Palermo districts (30 and 57 years old). WNV IgGs were also detected in three of these patients (two patients from Palermo and one from Trapani). IgG ELISA-positive samples were confirmed at the ISS using a specific plaque reduction neutralization assay (PRNT) [26]. The remaining two IgM-positive patients (69 and 72 years old) were from the Trapani district. In both cases, WNV RNA was detected in blood, and in one case, it was also detected in urine samples. Unfortunately, one of the two viraemic patients died 41 days after the onset of symptoms. Lineage-specific qRT‒PCR identified WNV-L2 as the lineage responsible for infection in both of the latest cases [29]. In the 2023 epidemic season, serological investigations were conducted on 31 patients. Low anti-WNV IgM values in the absence of IgG were detected in three patients, whereas low values of IgM and IgG were detected in a single patient. In none of these cases were specific immunoglobulins confirmed by PRNT. A single case of WNND was confirmed and involved a 54-year-old immunocompromised man from Trapani who tested negative for IgG and IgM but positive for WNV RNA in whole blood and CSF samples on 11 August 2023. Unfortunately, it was not possible to obtain a WNV genome sequence of sufficient quality to define the lineage of the virus involved.

### WNV entomological and animal data

All animal samples and pools of mosquitoes collected between September and November 2022 were analysed at IZSSi. WNV IgMs were detected in 10 horses, nine from the Trapani district and one from the Palermo area. One horse from Palermo that was IgM- and IgG-positive for WNV died 9 days after showing neurological symptoms. WNV IgG antibodies were also detected in a second horse from Palermo (2/22, 9.09%, 95% CI 2.9–21.10%), 23 horses (30.26%, 95% CI 19.93–40.59%), 10 birds (15.15%, 95% CI 6.50–23.80%) and five dogs (45.45%, 95% CI 16.03–74.88%) from the Trapani area. All IgG ELISA-positive samples were confirmed by a serum neutralization (SN) assay carried out at IZS-Te [28]. WNV RNA was not detected in any of the collected blood samples (Table 2). WNV-L2 RNA was instead detected in the spinal cord of a horse from Palermo that died after showing WNV clinical signs.

**Table 2:** **WNV animal surveillance in western Sicily in the 2022 transmission season**.

Trapani district				
Species	Number of samples	Anti-WNV IgM Positive	Anti-WNV IgG Positive	WNV RNA Positive (Blood)
Horse	76	9 (11.84%)	23 (30.26%)	0 (0%)
Chicken	66	ND	10 (15.15%)	0 (0%)
Dog	11	ND	5 (45.45%)	0 (0%)
Total	153	9 (5.88%)	38 (25.85%)	0 (0%)

Palermo District				
Species	Number of samples	Anti-WNV IgM Positive	Anti-WNV IgG Positive	WNV RNA Positive (Blood/Tissues)
Horse	22	1 (4.54%)	2 (9.09%)	1 (4.54%) Spinal Cord

*ND* not determined

Among the collected mosquitoes, *Culex pipiens* (48.72%, 95% CI 33.03–64.40%) and *Aedes albopictus* (41.02%, 95% CI 25.59–56.46%) were the most represented species, followed by *Culiseta longiareolata* (7.69%, 95% CI 0.67–16.05%) and *Ochlerotatus caspius* (2.56%, 95% 95% CI 2.39–7.52%) (a single individual collected in the Trapani district). All pools were negative when tested for the presence of WNV-L1 and WNV-L2 RNA by WNV-L1- and WNV-L2-specific qRT‒PCR [29] (Table 3).

**Table 3:** **Results of entomological surveillance in western Sicily in 2022**.

	Number of pools	*Culex pipiens*	*Aedes albopictus*	*Culiseta longiareolata*	*Ochlerotatus caspius*	WNV presence
Trapani District	35	16	15	3	1	0
Palermo District	4	3	1	0	0	0
Total	39	19 (48.72%)	16 (41.02%)	3 (7.69%)	1 (2.56%)	0

Mosquito identification and determination of WNV presence in female pools by real-time RT‒PCR.

Additionally, in the 2023 epidemic season, *Aedes albopictus* (44.44%, 95% CI 25.70–63.19%) and *Culex pipiens* (37.04%, 95% CI 18.82–55.25%) were the most represented species. *Anopheles maculipennis* (11.11%, 95% CI 0.74–22.96%) and *Culiseta longiareolata* (7.40%, 95% CI 2.47–17.29%) were also found. When the presence of WNV-L1 and WNV-L2 RNA was tested by specific qRT‒PCR [29], a *Culex pipiens* pool of 12 mosquitoes caught in the Trapani district in August was found to be positive for WNV-L2 RNA (Table 4).

**Table 4:** **Results of entomological surveillance in western Sicily in 2023**.

	*Culex pipiens*	*Aedes albopictus*	*Anopheles maculipennis*	*Culiseta longiareolata*	Total
Number of pools	10 (37.04%)	12 (44.44%)	3 (11.11%)	2 (7.40%)	27
WNV presence	1	0	0	0	1 (3.7%)

Mosquito identification and determination of WNV presence in female pools by real-time RT‒PCR.

## Discussion

The years 2022 and 2023 have been characterized by a high number of WND cases in Europe and Italy. As a vector-borne disease, WND occurrence is strongly influenced by environmental factors such as temperature, seasonal cycles, and the amount of rainfall [30]. In recent years, ecological and meteorological conditions, such as low winter rains and high spring temperatures, have probably favoured the reproduction of mosquitoes, increasing their numbers [31–33]. A greater abundance of mosquitoes increases the likelihood of mosquito bites, which in turn increases the transmission rate and consequently the circulation of WNV [34, 35]. In this study, we described the emergence and spread of WNV in western Sicily during the 2022 and 2023 transmission seasons in Trapani and nearby Palermo districts. Five WNND-confirmed human cases occurred in Sicily in 2022. All patients recovered from the illness except for one, who died 41 days after the onset of symptoms. Even if WNND generally represents only 1% of cases, fatality rates in patients with neuroinvasive illness are as high as 17% [36]. Following the occurrence of the human cases, the national surveillance program activities already in place intensified in the outbreak areas. IgM and WNV-L2 RNA detection in horses confirmed WNV circulation in the same areas where human cases were reported. The mild clinical course with low viraemia observed in most animals and the low occurrence of WNND cases and deaths observed in humans confirm that the severity of the clinical manifestation of WNV depends on the immune status of the host. The observation of a case of WNND in 2023 involving a man who was 54 years of age with chronic lymphatic leukaemia, fever and encephalitis confirmed the presence of WNV in Sicily and its ability to be related to severe cases of disease in humans. Unfortunately, since genome sequence analyses were not available, we could not determine whether the same 2022 strain was involved in the 2023 WNND case. However, in conjunction with the case of WNND in humans, in 2023, we demonstrated the presence of WNV-L2 in insects, but we did not detect symptomatic cases in equids. Our findings show that the WNV-L2 strains circulating in western Sicily from 2022 are capable of determining severe clinical presentations in humans and animals, as already described in Central Europe and other Mediterranean regions [16, 37].

Following the occurrence of human cases, preventive actions have been taken to reduce the number of mosquitoes and the degree of virus circulation in affected areas, together with health communication campaigns to increase awareness of the risk posed by exposure to mosquitoes. Competent authorities implemented a special mosquito control program using adulticides and larvicides in response to WNV vector abundance alerts. These actions might have contributed to preventing the occurrence of further WND cases in these areas. The National Blood Centre has also been informed and requested WNV testing of donors depending on the residence area or travel history. No Sicilian blood donors tested positive during the 2022 and 2023 WND outbreaks, unlike other regions of the national territory, where 160 blood donors tested positive from checks on blood bags. The region where the most positive donors were found was Lombardy, followed by Emilia-Romagna, Veneto and Piedmont [25, 38].

WNV RNA, which was collected mainly in the Trapani district, was not detected in either birds or mosquitoes sampled in 2022. This finding might indicate that WNV did not circulate in the surveyed area at the time of sampling. However, positive WNV serology in birds confirmed that WNV has circulated in the recent past. The findings of WNV-neutralizing antibodies in dogs living in the city of Trapani indicate that WNV is circulating or has circulated in urban areas, where the risk of infection in humans is relatively high. This result underlines once again the role that dogs might play during the vector season. In fact, sharing the domestic environment with humans can indicate WNV circulation in urban and suburban areas even before the onset of human cases in the population and could be used as a useful tool for preventing WNV human infections [14, 16, 39, 40]. Although WNV RNA was not detected in the pools of female mosquitoes tested in 2022, the occurrence of horse cases and the WNV-positive serology found in the screened animals living in the same areas of human cases proved that in those areas, suitable habitats for WNV vectors were available and that WNV circulation was possible. In contrast, in 2023, positivity in a pool of *Culex pipiens* in the absence of symptoms in horses was useful for confirming the circulation of the virus and its involvement in WNND in humans.

Surveillance of avifauna should be intensified both on resident birds belonging to “synanthropic” species, both on rural and outdoor poultry farms, and on migratory birds. The latter, during the migration period, could play a crucial role because it could be responsible for introducing the virus into resting or nesting areas located along migratory routes. To this end, they should have involved the Wild Animal Recovery Centres, the Forestry Corps and other Territorial and Environmental Institutions. The timely detection of viruses circulating in wild populations is useful for preventing the circulation of these viruses in domestic poultry farms, where WNV finds ideal conditions for transmission. Controls could be implemented even through the positioning of groups of sentinel birds preferably located near areas where wild avifauna and/or wetlands (fresh or brackish water collections of any type) are concentrated. It is also necessary to fully understand the different immune responses of the hosts, as both L1 and L2 lineages infect the same bird species but may have different outcomes in different species, leading to the fact that most human outbreaks worldwide are caused by L1 strains despite the strong presence of L2 strains [20]. In consideration of the current epidemiological scenario, it would also be appropriate to strengthen entomological surveillance, increasing the number of fixed traps for catching insects, identifying new areas where environmental microclimatic conditions favourable to the development of the vectors are created, and/or with a high concentration of wild birds. Entomological surveillance in WNV risk areas, such as Sicily, aims to define the composition of the culicid fauna in these areas. As part of entomological surveillance, monitoring of vectors should also be carried out with the positioning of traps at critical points of entry (PoE), including airports and ports, to identify new species of invasive mosquitoes (e.g., *Aedes aegypti*). A further surveillance approach could take advantage of citizens'help through the “Mosquito Alert app” (https://www.mosquitoalertitalia.it/), an app that can be considered a “citizen science project” that allows the collection of invaluable information on biodiversity and invasive mosquito species. It requires that photographic records of mosquitoes made by citizens be sent to expert entomologists who identify the species, compile a database, and map the spread [41].

The results of this study provide a picture of the 2022–2023 WNV arthropod vector season in Sicily, confirming the WNV-L2 circulation for the first time in western Sicily and supporting the predicted suitable conditions for the spread of WNV in Italy, with a progressive increase in WND-L2-affected areas [34]. Some recent phylogenetic analyses revealed that the 2022 WNV outbreaks in western Sicily were linked to a novel WNV-L2 strain from the Balkans, highlighting the importance of continuous molecular surveillance to detect viral circulation early throughout the country to monitor the arrival and evolution of new variants, whose potential virulence can be predicted on the basis of the genetic similarity of the strains [35, 36].

Close collaboration between veterinary and medical institutions has facilitated the detection and identification of WNV-L2 as the virus responsible for the WND outbreak and has favoured its control. Once WNV has established in a settled area, the risk of maintenance and diffusion of the virus is high, and only integrated surveillance systems, combining vector control, risk communication, adoption of individual protection measures, and screening of blood and blood components, organ, and tissue donations, can improve the ability to detect, prevent and reduce the transmission of WNV to humans and animals via a One-Health approach. Integrating viral genetic data from human and animal WNV strains with information on the movements of migratory birds and the susceptibility of various species to infection could lead to a deeper understanding of how the virus spreads. Fundamental information to predict and mitigate the impact of future epidemics constitutes a study model for other emerging viruses.

## Data Availability

The data that support the fndings of this study are available from the corresponding author upon reasonable request. Source data are provided with this paper.
